# Acetyl Co-A Carboxylase Inhibition Halts Hyperglycemia Induced Upregulation of De Novo Lipogenesis in Podocytes and Proximal Tubular Cells

**DOI:** 10.3390/metabo12100940

**Published:** 2022-10-03

**Authors:** Pradeep Kayampilly, Nancy Roeser, Thekkelnaycke M Rajendiran, Subramaniam Pennathur, Farsad Afshinnia

**Affiliations:** 1 Department of Internal Medicine-Nephrology, University of Michigan, Ann Arbor, MI 48105, USA; 2 Michigan Regional Comprehensive Metabolomics Resource Core, University of Michigan, Ann Arbor, MI 48105, USA; 3 Department of Pathology, University of Michigan, Ann Arbor, MI 48109, USA; 4 Department of Molecular and Integrative Physiology, University of Michigan, Ann Arbor, MI 48109, USA

**Keywords:** diabetes, de novo lipogenesis, acetyl Co-A carboxylase, palmitate, podocytes, tubules, lipid metabolism

## Abstract

The effect of glycemic stress on de novo lipogenesis (DNL) in podocytes and tubular epithelial cells is understudied. This study is aimed (A) to show the effect of glycemic stress on DNL, and (B) to assess the effect of acetyl-Co A (ACC) inhibition on halting upregulation of DNL, on the expression of other lipid regulatory genes in the DNL pathway, and on markers of fibrosis and apoptosis in podocytes and tubular epithelial cells. We used cultured mouse primary tubular epithelial cells, mouse proximal tubular (BUMPT) cells, and immortal mouse podocytes and measured their percentage of labeled ^13^C_2_-palmitate as a marker of DNL after incubation with ^13^C_2_ acetate in response to high glucose concentration (25 mM). We then tested the effect of ACC inhibition by complimentary strategies utilizing CRISPR/cas9 deletion or incubation with *Acaca* and *Acacb* GapmeRs or using a small molecule inhibitor on DNL under hyperglycemic concentration. Exposure to high glucose concentration (25 mM) compared to osmotic controlled low glucose concentration (5.5 mM) significantly increased labeled palmitate after 24 h up to 72 h in podocytes and primary tubular cells. Knocking out of the ACC coding *Acaca* and *Acacb* genes by CRISPR/cas9, downregulation of *Acaca* and *Acacb* by specific antisense LNA GapmeRs and inhibition of ACC by firsocostat similarly halted/mitigated upregulation of DNL and decreased markers of fibrosis and programmed cell death in podocytes and various tubular cells. ACC inhibition is a potential therapeutic target to mitigate or halt hyperglycemia-induced upregulation of DNL in podocytes and tubular cells.

## 1. Introduction

Diabetes is the leading cause of end stage kidney disease (ESKD) in the United States [1] and in many parts of the world [2]. It causes significant patient and societal burdens. In 2018, the Medicare fee-for-service expenditure for the care of ESKD increased to $49.2 billion [3], a cost which was more than the entire budget of the National Institute of Health for the fiscal year 2018 [4] and was driven by an increasing number of patients with diabetes. Various projections report that the prevalence of ESKD will continue to increase by 44% in the year 2030 [5,6,7,8], mainly owing to the continued rise in the prevalence of diabetes. To mitigate the patient and societal burden of kidney failure the underpinning mechanisms responsible for disease progression should be halted.

Diabetic nephropathy is a common complication in human type 1 and 2 diabetic patients. Early changes in glomeruli including podocyte dysfunction are critical for the subsequent development of glomerulosclerosis [9]. Podocytes are highly specialized terminally differentiated cells that cover the urinary side of the glomerular basement membrane (GBM). Together with glomerular endothelial cells, podocytes are responsible for the maintenance of the GBM, its charge barrier, and the shape and integrity of the glomerular capillary loop [9,10]. Proximal renal tubular epithelial cells are highly metabolically active cell lines that receive the glomerular filtrate from the Bowman capsule and are responsible for up to 60% reabsorption of glomerular filtrates. In diabetes, both podocytes and tubular epithelial cells undergo dysfunction and maladaptive changes that lead to glomerulosclerosis and tubulointerstitial fibrosis. Some of the mechanisms involved in the pathogenesis of diabetes complication include hyperglycemic mediated upregulation of diacylglycerol-protein kinase C activation, increased sorbitol-myoinositol flux, redox alterations, increased glycemic advanced end-products, hexosamine biosynthesis, and dyslipidemia among others [11]. Diabetic dyslipidemia by traditional measures is characterized as increased plasma triglycerides and LDL-cholesterol, in the context of decreased HDL-cholesterol [11]. However, complex dyslipidemia in diabetes is not adequately explained by traditional measures. In a study of native American Indians with type 2 diabetes, we showed upregulation of renal acetyl-Co A carboxylase (ACC) encoding genes in association with increased circulating di- and triacylglycerols in progressors of diabetic kidney disease coupled with markers of impaired β-oxidation, suggesting that upregulation of de novo lipogenesis (DNL) might be an underpinning mechanism of diabetic kidney disease progression at an early stage [12].

In DNL, carbohydrates are converted to fatty acids through a highly regulated process [13]. DNL is dependent on the activity of 3 enzymes: ATP citrate lyase (ACLY), ACC, and fatty acid synthetase (FASN) [12,13]. ACLY converts cytosolic citric acid to acetyl-Co A the precursor of Malonyl-Co A. ACC is the key regulator of DNL, is influenced by insulin, glucagon, and epinephrine, and consists of two isoforms ACC1 and ACC2 [14]. ACC1 is encoded by *Acaca*, and ACC2 is encoded by the *Acacb* gene. ACC1 is a cytosolic enzyme committed to the rate-limiting step of DNL and has a high abundance in the liver, adipose tissues, and mammary glands (14). ACC2 is a mitochondrial-membrane-associated enzyme that generates malonyl-CoA and inhibits carnitine palmitolytransferase-1, which is responsible for long-chain fatty acid transport into mitochondria and has a high abundance in oxidative tissues such as the heart and skeletal muscles [15,16]. ACC activation on one hand leads to malonyl-Co A mediated inhibition of carnitine palmitoyltransferase-1 (CPT1), reducing fatty acid β-oxidation, and on the other hand, upregulates the FASN complex promoting fatty acid synthesis via DNL [12,13]. Under normal physiologic conditions liver and adipose tissue are the major sites of DNL [13,17]. However, our observation of renal *ACACA* expression correlated with circulating markers of DNL in native American Indians [12] suggests that under hyperglycemic stress DNL can be upregulated in the kidney. Increased flux of free fatty acids can result in overactivity of membrane diacylglycerol/protein kinase-C [18,19] and inhibitor of nuclear factor kappa B kinase and nuclear factor kappa B [20,21], activation of AMP-activated protein kinase and mammalian target of rapamycin complex-1 signaling pathways, induces insulin resistance, promotes mitochondrial superoxide generation and endoplasmic reticulum stress, impairs the podocyte actin cytoskeleton, induces autophagy, and eventually triggers apoptosis and cell death [22,23,24,25,26,27,28,29]. As such hyperglycemia may pose detrimental effects on markers of cell death and apoptosis (*Fas*) and fibrosis (*Col1a1, Col3a1, Col6a1, Tgfb1*). Hence, upregulation of renal DNL by hyperglycemia may be a distinct mechanism of diabetic kidney disease progression. However, the effect of lipogenesis in kidneys, especially in podocytes, is understudied [30,31,32], so further studies are required to demonstrate the importance of ACC-mediated lipogenesis in podocytes. In this study, our aims are (A) to show the causal effect of glycemic stress on the upregulation of DNL in podocytes and tubular epithelial cells, two principal cell types involved in metabolic response to a diabetic state; (B) to demonstrate the downregulation of DNL by ACC manipulation using various means including CRISPR/cas9 mediated *Acaca* and *Acacb* knocking down (KO), oligonucleotide mediated gene silencing, and ACC pharmacological inhibitor; and (C) to provide more insight on the effect of ACC blockade on markers of fibrosis (*Col1a1, Col3a1, Col6a1, Tgfb1*), on the expression of other lipid regulatory genes (*Acly, Elovl6, Scd1, Pnpla3, Srebp1c, Gpat, Pltp, G6pd, and Dgat*), and on the upstream regulator of cell death and apoptosis (*Fas*). We hypothesize that high glucose concentration increases the percentage of labeled palmitate as a marker of enhanced DNL and that ACC inhibition halts the hyperglycemic-mediated upregulation of DNL and decreases gene expression of fibrotic markers and cell death in the podocytes and proximal tubular cell lines under hyperglycemic conditions.

## 2. Results

### 2.1. Hyperglycemia Upregulates De Novo Lipogenesis in Healthy Podocytes and Proximal Epithelial Cells In Vitro

**Figure 1:**
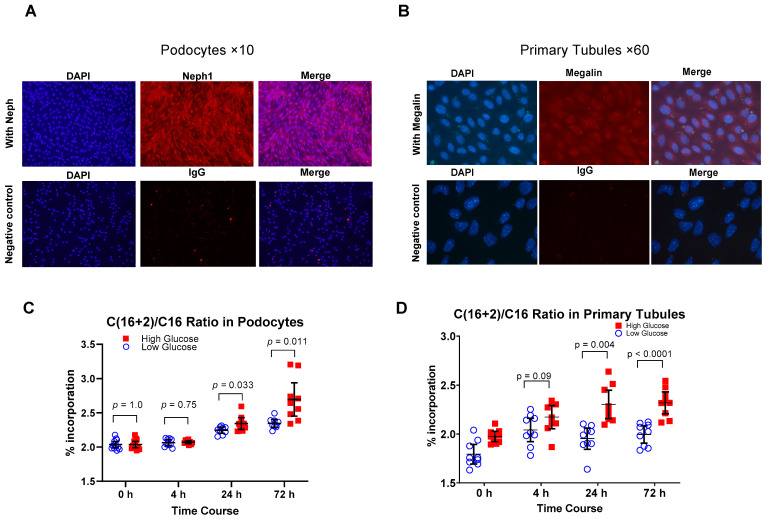
Upregulation of DNL with hyperglycemia in podocytes and primary tubular cells. Immuno-staining with cell-specific markers was used to confirm podocytes and primary tubular cells, Showing NEPH1 staining for podocytes (**A**) and megalin for primary tubular epithelial cells (**B**). Increased percentage of labeled palmitate with exposure to high glucose concentration (25 mM) at 24 and 72 h compared to low glucose concentration (5.5 mM) media in podocytes (**C**) and primary tubular cells (**D**) indicates upregulation of de novo lipogenesis by high glucose concentration. Number of experiments is 3, each time in triplicates for (**C**,**D**). Values are mean and 95% confidence interval.

To demonstrate the cell specificity of the cultured cells we showed NEPH1 marker on podocytes (Figure 1A) and megalin on the primary tubular epithelial cells (Figure 1B) using immunofluorescence. To demonstrate the upregulation of DNL; using mass spectrometry, we compared the percentage incorporation of ^13^C_2_-sodium acetate in construct of newly synthesized palmitate by measuring the ratio of C16+2 labeled palmitate to C16 palmitate at 0, 4, 24, and 72 h after exposure of the cell lines to low (5.5 mM) and high (25 mM) glucose media in vitro. We found that exposure to high glucose significantly increased the percentage of the labeled palmitate in podocytes after 24 h (*p* = 0.033) and to a greater extent after 72 h (*p* = 0.011) as compared to exposure to euglycemic conditions (Figure 1C). Similarly, we found that exposure to high glucose increased the ratio of labeled palmitate after 24 (*p* = 0.004) and 72 h (*p* < 0.001) in primary tubular epithelial cells in vitro (Figure 1D). These findings indicate that high glucose concentration upregulates DNL in podocytes and tubular epithelial cells in vitro.

### 2.2. Acetyl-Co A Carboxylase Encoding Gene Deletion Halts Hyperglycemia Mediated Upregulation of De Novo Lipogenesis in Podocytes and BUMPT Cells In Vitro

To establish the mechanism of halting DNL, we investigated the effect of *Acaca* and *Acacb* knock-down on halting the upregulation of DNL in podocytes and BUMPT cells. First, we knocked out *Acaca* and *Acacb* genes separately and together in podocytes and BUMPT cells in vitro using CRISPR-Cas9 gene editing technology. 

**Figure 2:**
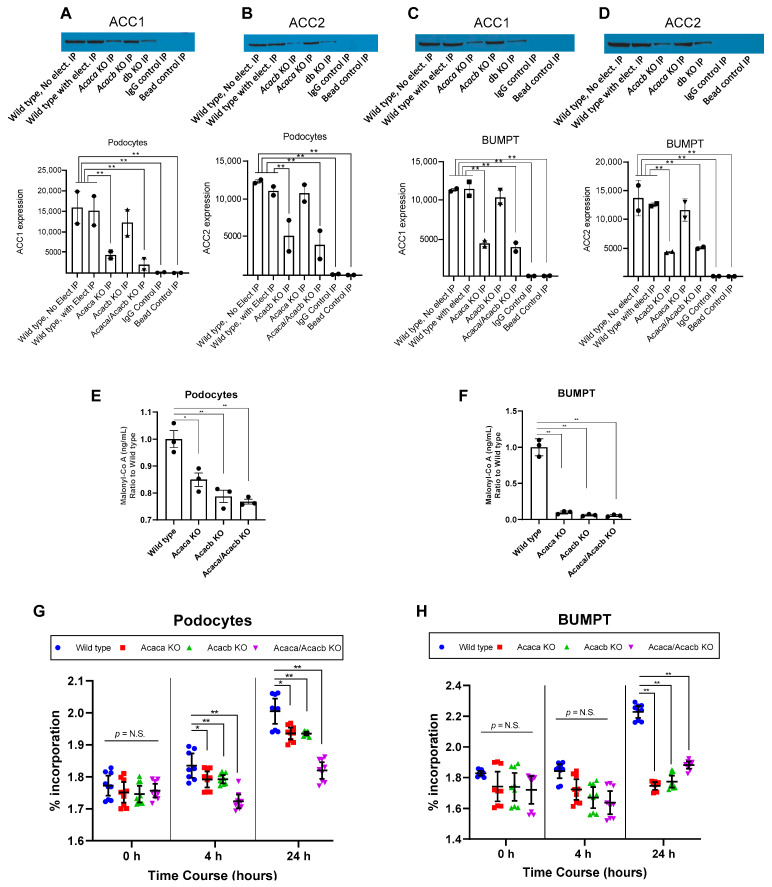
Acaca and Acacb knocking out using CRISPR-Cas9 halts glycemic mediated upregulation of DNL in podocytes and BUMPT cells in vitro. Measurement of protein expression by immunoprecipitation showed that Acaca KO and Acaca/Acacb double KO decreased expression of ACC1 protein in podocytes (**A**). Acacb KO and Acaca/Acacb double KO decreased expression of ACC2 protein in podocytes (**B**). Acaca KO and Acaca/Acacb double KO decreased expression of ACC1 protein in BUMPT cells (**C**). Acacb KO and Acaca/Acacb double KO decreased expression of ACC2 protein in BUMPT cells (**D**). For immunoprecipitation blots, wild-type cells with and without electroporation were used as positive controls, and IgG and control beads were used as negative loading controls (**A**–**D**). Acaca and Acacb gene KO in podocytes (**E**) and BUMPT cells (**F**) cultured in high glucose concentration (25 mM) suppressed production of malonyl-Co A compared to wild-type cells. KO mitigated the time dependent increase in percentage of labeled palmitate exposed to high glucose concentration (25 mM) compared with wild-type podocytes (**G**) and BUMPT cells (**H**) * *p* < 0.05, ** *p* ≤ 0.01. Values are mean and standard errors. Number of experiments is 2 in (**A**) to d, 3 in (**E**,**F**), and 3 with each experiment in triplicates in (**G**,**H**).

Next, we measured the expression of ACC1 and ACC2 proteins in podocytes and BUMPT cells. We applied wild-type cells with and without electroporation as positive controls. In podocytes, *Acaca* KO or double KO (DKO), but not *Acacb* KO significantly reduced expression of ACC1 protein (Figure 2A), while *Acacb* KO or double KO significantly reduced expression of ACC2 protein (Figure 2B) compared to wild-type cells. In BUMPT cells, *Acaca* KO or double KO, but not *Acacb* KO significantly reduced the expression of ACC1 protein (Figure 2C), while *Acacb* or double KO, but not *Acaca* KO significantly reduced the expression of ACC2 protein (Figure 2D). These findings suggest that KO of acetyl-co A carboxylase encoding genes halts expression of the corresponding ACC protein in podocytes and BUMPT cells in vitro. 

Next, we measured malonyl-co A concentration as a marker of ACC activity in KO cells 48 h after exposure to high glucose media (25 mM) and showed its significant reduction by *Acaca* KO, *Acacb* KO, or their double KO in podocytes (Figure 2E), and BUMPT cells (Figure 2F). These findings suggest that *Acaca* and *Acacb* KO reduces the activity of ACC enzymes. 

We then compared the abundance of newly synthesized palmitate in ACC deficient cells with the wild-type cells by measuring the percentage of labeled palmitate in podocytes and BUMPT cells following exposure to labeled acetate in vitro. In podocytes, there was not any difference in the percentage of labeled palmitate between various groups at time 0, however, there was a graded increase in the percentage of labeled palmitate from time 0 to 24 h in wild-type cells. On the other hand, the increase in labeled palmitate in *Acaca* KO, *Acacb* KO, or double KO at 4 and 24 h was attenuated or completely halted, so that at 24 h the percentage of labeled palmitate in wild-type podocytes was significantly higher than that in *Acaca* KO, *Acacb* KO, or double KO podocytes (Figure 2G). Similarly, in BUMPT cells, while there was not any difference in the percentage of labeled palmitate between various groups at time 0, and 4 h, there was a significant increase in the percentage of labeled palmitate from time 0 to 24 h in wild-type cells. On the other hand, there were no significant changes in the percentage of labeled palmitate in KO cells over time, so at 24 h the percentage of labeled palmitate in wild-type BUMPT cells was significantly higher than that in *Acaca* KO, *Acacb* KO, or double KO cells (Figure 2H). These findings indicate that KO of acetyl-co A carboxylase encoding genes halts hyperglycemia-mediated upregulation of DNL in podocytes and BUMPT cells in vitro.

### 2.3. Alteration of mRNA Markers of Other Genes by Acaca and Acacb KO

**Figure 3:**
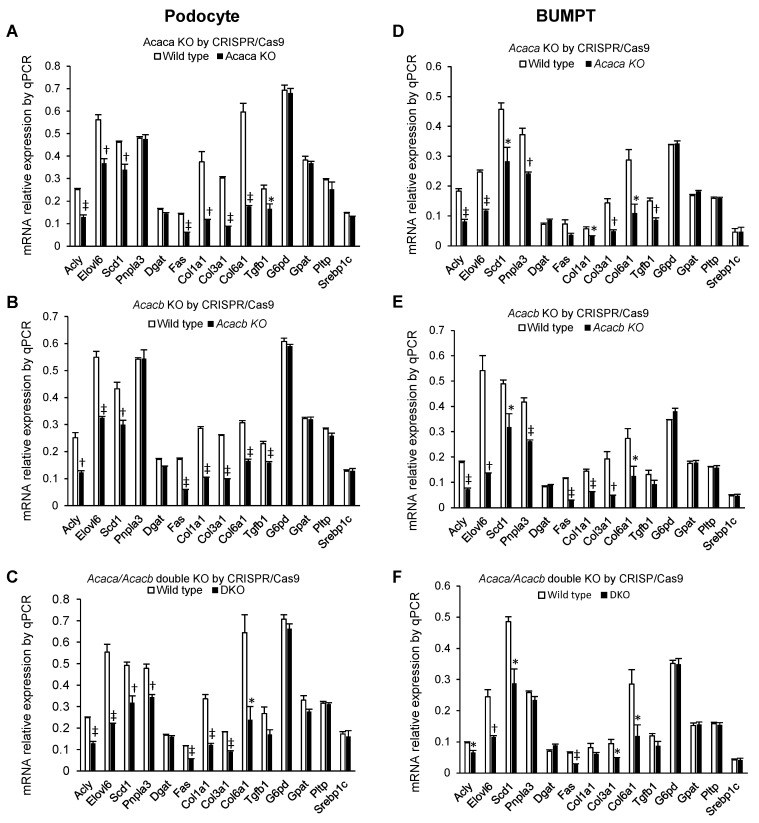
Comparing mRNA relative expression of gene markers in Acaca KO, Acacb KO, and Acaca/Acacb double KO using CRISPR/Cas9 in podocytes (**A**–**C**) and BUMPT cells (**D**–**F**) compared with wild-type cell lines. Values are mean and standard error in triplicate. * *p* < 0.05, † *p* < 0.01, ‡ *p* < 0.001. Number of experiments is 3 in all panels.

As compared to the control wild-type cells, expression of DNL-related genes *Acly*, *Elovl6*, and *Scd1*; apoptosis regulator *Fas;* and markers of fibrosis *Col1a1*, *Col3a1*, and Col6a1 were significantly suppressed in *Acaca* KO, *Acaca* KO, and *Acaca*/*Acacb* double KO podocytes (Figure 3A–C). Expression of Pnpla3 was lower in double KO, but the lower expression of *Tgfb1* did not reach statistical significance. There were no changes in the expression of *G6pd*, *Gpat*, *Pltp*, *Dgat* and *Srebp1c* (Figure 3A–C). In BUMPT cells *Acaca*, *Acacb*, and *Acaca*/*Acacb* double KO genes decreased the expression of DNL-related genes *Acly*, *Elovl6*, and *Scd1*; markers of fibrosis *Col3a1*, *Col6a1* as compared to wild-type cells except that the lower Fas expression in *Acaca* KO, lower *Tgfb1* in *Acacb* KO and double KO, and lower *Col1a1* in double KO did not reach statistical significance (Figure 3D–F).

### 2.4. Down Regulation of Glycemic Mediated De Novo Lipogenesis by GapmeRs in Podocytes and Primary Tubular Epithelial Cells In Vitro

**Figure 4:**
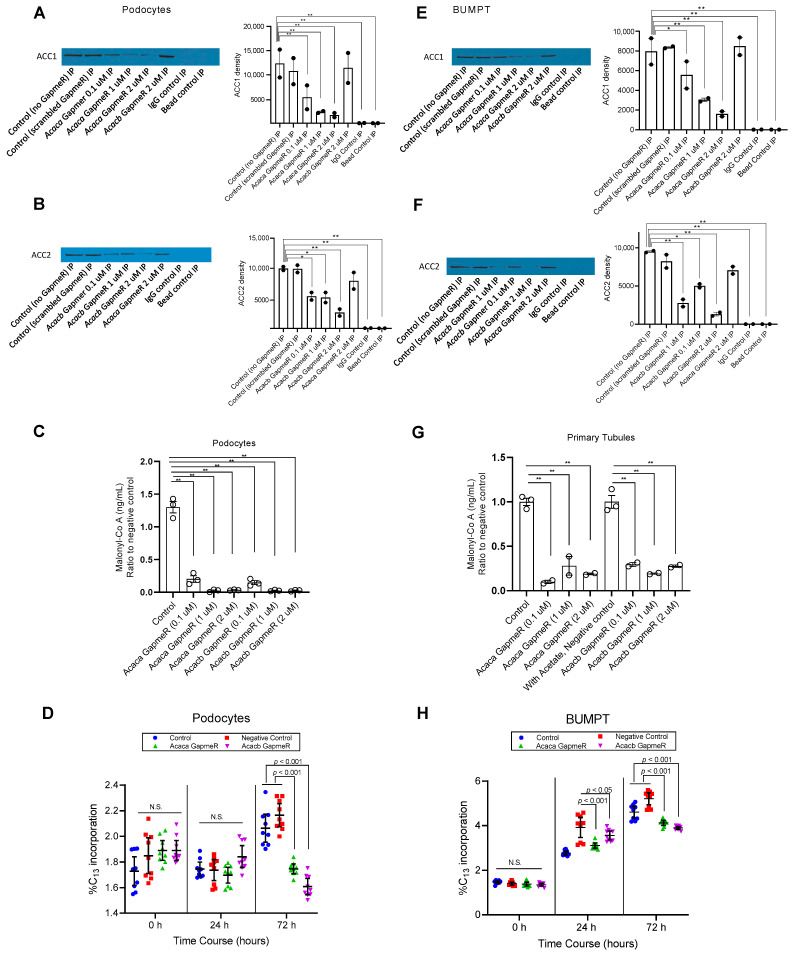
Down regulation of glycemic mediated DNL by Acaca and Acacb GapmeRs in podocytes and primary tubular epithelial cells in vitro: Measurement of protein expression by immunoprecipitation showed that Acaca-specific GapmeR decreased expression of ACC1 protein in a dose-dependent manner in podocytes (**A**) and primary tubular epithelial cells (**E**) compared to control cells. Acacb-specific GapmeR decreased expression of ACC2 protein in a dose-dependent manner in podocytes (**B**) and primary tubular epithelial cells compared to control cells (**F**). In (**A**,**B**,**E**,**F**) positive loading controls are cells with no exposure to GapmeR and cells with exposure to scrambled gapmeR. The negative controls are IgG and Bead controls. Exposure to GapmeR showed a dose-dependent suppression of malonyl-Co A activity in podocytes (**C**) and primary tubular epithelial cells (**G**) treated by targeting Acaca and Acacb genes compared to control cells cultured in high glucose concentration (25 mM) for 48 h. Exposure to GapmeR mitigated time-dependent increase in the percentage of labeled palmitate in high glucose concentration (25 mM) over time as compared to control cells in podocytes (**D**) and in BUMPT cells (**H**). * *p* < 0.05, ** *p* ≤ 0.01. Values are mean and standard errors. Number of experiments is 2 in (**A**,**B**,**E**,**F**); 3 in (**C**), 3 in controls of (**G**), and 2 in other conditions of (**G**). Number of the experiment is 3 in (**D**,**H**) with each experiment in triplicate.

We utilized a complementary strategy to the CRISPR/Cas9 to demonstrate the potential of ACC inhibition on down regulation of hyperglycemia-induced upregulation of DNL. We compared the ACC1/2 expression and malonyl-co A concentration in response to various doses of GapmeRs. In podocytes, ACC1 expression was significantly reduced by *Acaca* (not *Acacb*) GapmeR in a dose-dependent manner compared to control and negative control (*p* < 0.001, Figure 4A). Similarly, there was a significant reduction in expression of ACC2 protein in a dose-dependent manner to *Acacb* (not *Acaca*) GapmeR compared to control or negative control (*p* < 0.01, Figure 4B). In Primary tubular cells, exposure to *Acaca* (not *Acacb*) GapmeR significantly reduced ACC1 expression dose dependently compared to control and negative control (*p* < 0.01, Figure 4E), while exposure to *Acacb* (not *Acaca*) GapmeR significantly reduced the expression of ACC2 protein dose-dependently when compared with control and negative control group (Figure 4F). These findings suggest that Gapmer downregulation of *Acaca* and *Acacb* genes can reduce the expression of the corresponding proteins in podocytes and primary tubular epithelial cells in vitro.

Next, we compared malonyl Co-A concentration by study groups. In podocytes, exposure to *Acaca* and *Acacb* GapmeRs significantly reduced malonyl-CoA production in a dose-dependent manner compared with negative control after 48 hours from exposure to high (25 mM) glucose media (*p* < 0.0001, Figure 4C). Similarly, in primary tubular cells, exposure to *Acaca* and *Acacb* GapmeRs significantly reduced malonyl-Co A production compared to negative control 48 hours after exposure to high (25 mM) glucose media (*p* < 0.0001, Figure 4G). These findings suggest that GapmeR mediated downregulation of *Acaca* or *Acacb* gene can mitigate the activity of the corresponding enzymes in podocytes and primary tubular epithelial cells in vitro. 

Next, we compared the percentage of the labeled palmitate as a marker of newly synthesized palmitate through DNL from 0 h to 72 h after exposure of podocytes (Figure 4D) and BUMPT cells (4H) to high glucose concentration compared to control groups. Accordingly, the control groups showed a significant increase in the percentage of labeled palmitate from 0 h to 72 h, so their levels were significantly higher than the groups exposed to GapmeRs in podocytes (Figure 4D) and BUMPT cells (Figure 4H). 

### 2.5. Alteration of mRNA Markers of Other Genes in GapmeR Mediated Acaca and Acacb Downregulated Cells

**Figure 5:**
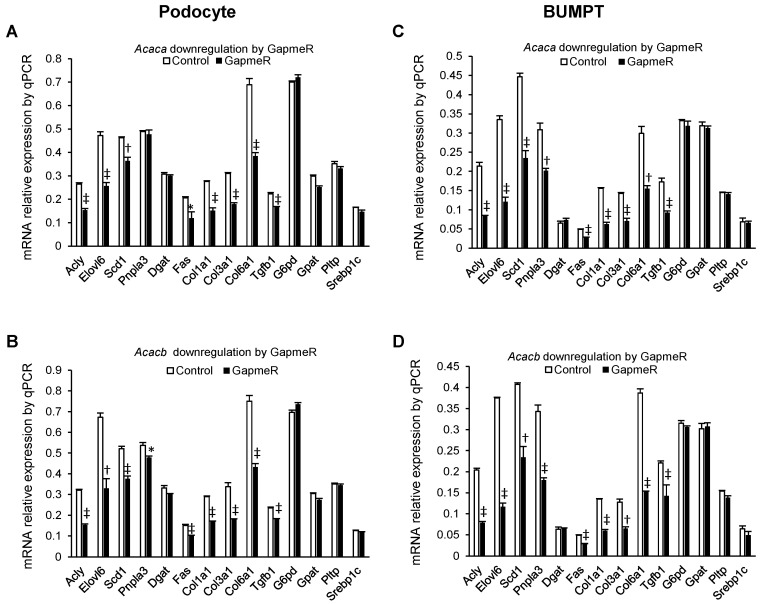
Comparing mRNA relative expression of gene markers in podocytes (**A**,**B**) and BUMPT cells (**C**,**D**) exposed to *Acaca* and *Acacb* GapmeRs (2 uM) compared with the negative control group. Values are mean and standard error in triplicate. * *p* < 0.05, † *p* < 0.01, ‡ *p* < 0.001. Number of experiments is 3 in all panels.

As compared to negative controls, exposure to *Acaca* and *Acacb* Gapmers led to a significantly decreased expression of *Acly, elovl6*, *Scd1*, *Fas*, *Col1a1*, *Col3a1*, *Col6a1*, and *Tgfb1* in podocytes, but no changes in expression of *Dgat*, *Srebp1c*, *G6pd*, *Gpat*, and *Pltp* (Figure 5A,B). In podocytes *Acacb,* Gapmer also promoted decreased expression on *Pnpla3*. In BUMPT cells exposure to *Acaca* and *Acacb* GapmeRs led to decreased expression of *Acly, elovl6*, *Scd1*, *Pnpla3*, *Fas*, *Col1a1*, *Col3a1*, *Col6a1*, and *Tgfb1*, but no changes in expression of *Dgat, Srebp1c*, *G6pd*, *Gpat*, and *Pltp* (Figure 5C,D).

### 2.6. Down Regulation of Hyperglycemia Mediated De Novo Lipogenesis by Pharmacological ACC Inhibitor in Podocytes and Primary Tubular Epithelial Cells In Vitro

**Figure 6:**
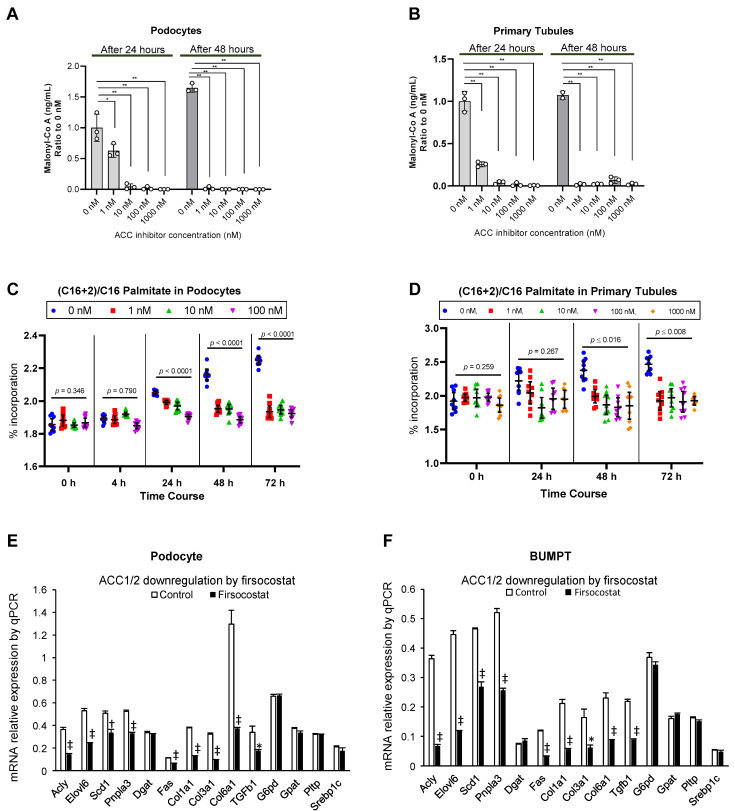
Down regulation of glycemic mediated DNL and change in expression of coregulatory genes by pharmacological ACC inhibitor in podocytes and primary tubular epithelial cells in vitro: Firsocostat exposure in podocytes (**A**) and primary tubular epithelial cells (**B**) cultured in high glucose concentration (25 mM) as compared to respective control cells showed a time and dose-dependent suppression of malonyl-Co A. Exposure to firsocostat halted increased percentage of labeled palmitate in high glucose concentration (25 mM) media over time compared to control cells in podocytes (**C**) and BUMPT cells (**D**) * *p* < 0.01, ** *p* < 0.001. Comparing the mRNA relative expression of gene markers in podocytes (**E**) and BUMPT cells (**F**) treated with firsocostat (1 µM) compared with control group. * *p* < 0.05, † *p* < 0.01, ‡ *p* < 0.001. Values are mean and 95% confidence interval in (**C**,**D**) and mean with standard error in other panels. Number of experiments is 3 in all panels with each experiment running in triplicates in (**C**,**D**).

To further demonstrate the potential of ACC inhibition on downregulation of hyperglycemia-mediated DNL, we examined pharmacologic inhibition of ACC. First, we exposed the podocytes in high (25 mM) glucose media to various doses of ACC inhibitor firsocostat at 0, 1, 10, 100, and 1000 nM concentrations. We found that after 24 h from exposure, there was a graded and dose-dependent decline in the level of malonyl-Co A compared to 0 nM, and after 48 h all doses suppressed the malonyl-Co A significantly and similarly (*p* < 0.01, Figure 6A). In primary tubular cells, we found a graded and dose-dependent decline in malonyl-Co A concentration compared to 0 nM after 24 h, and a similar and significant decline after 48 h in all groups compared with 0 nM. 

Next, we compared the abundance of newly synthesized palmitate in the cell lines exposed to various doses of firsocostat over time up to 72 h after exposure to high (25 mM) glucose media. In podocytes, there has not been any difference in percentage of labeled palmitate between various groups at 4 h between various doses of firsocostat. The abundance of labeled palmitate in 0 nM gradually and steadily increased from 24 to 72 h compared to other doses, while the abundance of labeled palmitate remained unchanged with exposure to any dose of firsocostat over time, so that the levels of palmitate in 0 nM was higher than those exposed to various doses of the inhibitor as 24 h and the highest at 72 h (*p* < 0.0001, Figure 6C). In primary tubular cells, there has not been any differences in labeled palmitate up to 24 h between various doses of firsocostat compared to no firsocostat. The level of labeled palmitate in cells exposed to no inhibitor increased at 48 and 72 h, while the level remained unchanged in other groups over time, so that the levels of palmitate in 0 nM was higher than those exposed to various doses of the inhibitor as 48 (*p* < 0.016) and 72 h (*p* < 0.008, Figure 6D). These findings suggest that pharmacological inhibition of ACC can mitigate DNL in podocytes and primary tubular epithelial cells in vitro.

### 2.7. Alteration of mRNA Markers of Other Genes by Pharmacological ACC Inhibitor

As compared to controls, use of ACC inhibitor firsocostat led to significantly decreased expression of *Acly, Elovl6, Scd1, Pnpla3, Fas, Col1a1, Col3a1, Col6a1*, and *Tgfb1*, but no changes in expression of *Dgat*, *Srebp1c*, *G6pd*, *Gpat*, and *Pltp* in podocytes as well as in BUMPT cells (Figure 6E,F).

### 2.8. Decreased Expression of Fibronectin with ACC Blockade

**Figure 7:**
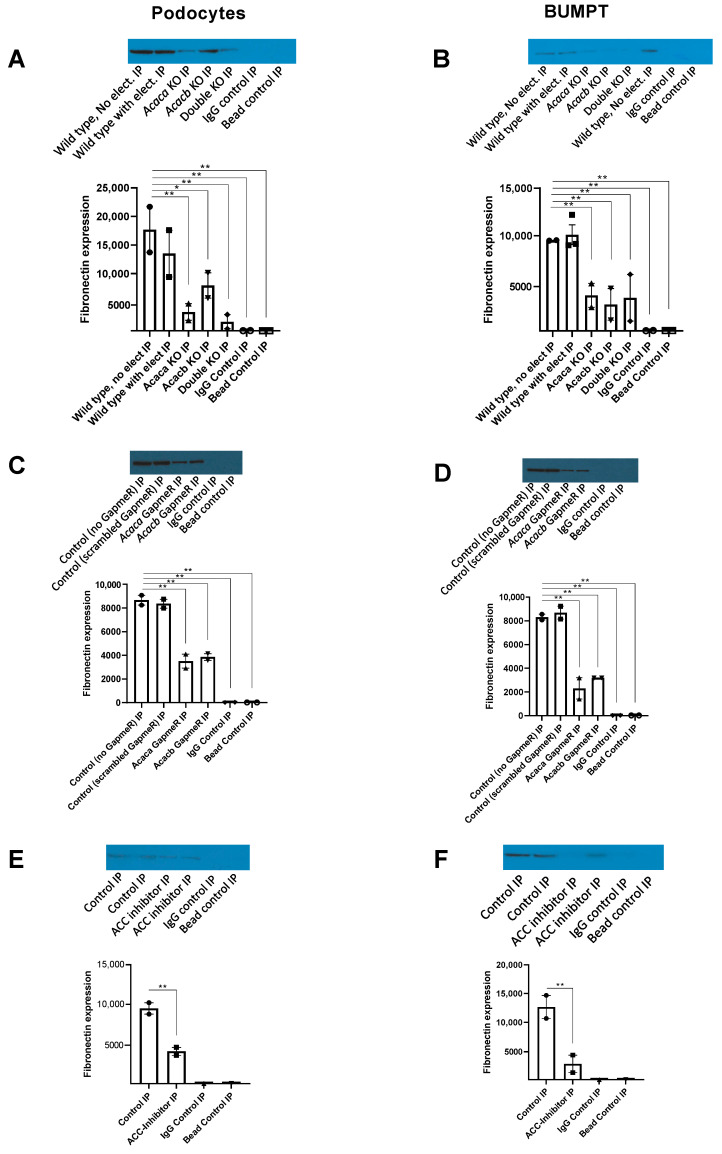
Decreased expression of Fibronectin with ACC blockade. Expression of fibronectin is diminished by knocking out of ACC1 and 2 encoding genes using CRISPR/Cas9 gene knock out in podocytes (**A**) and BUMPT cells (**B**); by exposure to *Acaca* and *Acacb* GapmeRs (2 uM) in podocytes (**C**) and BUMPT cells (**D**); and by exposure to firsocostat (2 uM) in podocytes (**E**) and BUMPT cells (**F**). Positive loading control in (**A**,**B**) are wild-type cells with and without electroporation, in panels (**C**,**D**) they are cells exposed to no GapmeR or scrambled GapmeR, and in (**E**,**F**) they are cells without exposure to ACC-inhibitor. In all panels (**A**–**E**), negative loading controls are IgG and bead controls. Number of experiments in all panels is 2 except for “Wild-type with electroporation” in (**B**) which had 3 experiments. For (**A**,**B**), wild-type cells with and without electroporation were used as two separate positive controls. Values are mean and standard error. * *p* < 0.05, ** *p* ≤ 0.01.

CRISPR/cas9 KO of *Acaca* and *Acacb* gene reduced expression of fibronectin in podocytes (Figure 7A) and BUMPT cells (Figure 7B). Similarly, downregulation of these genes by exposure to corresponding GapmeRs (2 uM) reduced expression of fibronectin in podocytes (Figure 7C) and BUMPT cells (Figure 7D). As well, exposure to 2 uM of theACC pharmacological inhibitor firsocostat diminished expression of fibronectin in these cells at 72 h (Figure 7E,F). 

## 3. Discussion

In this mechanistic study, we showed upregulation of DNL in proximal tubular epithelial cells and podocytes in response to high glucose concentration consistent with a prior report (30). We also showed that targeting ACC by various interventions including *Acaca* and *Acacb* gene KO/downregulation as well as ACC enzyme inhibition effectively halted/mitigated the hyperglycemia-induced upregulation of DNL and reduced gene markers of lipogenesis, fibrosis, and programmed cell death in tubular cells and podocytes. 

In a study on cultured human proximal tubular epithelial cells (HK-2) Xu et al. reported that exposure to high glucose (30 mmol/L) promoted the transition of the epithelial cells to mesenchymal cells, a process that involved an early-stage deposition of fatty acids, followed by accumulation of triglycerides, malonyl CoA, and lower fatty acid β-oxidation rate at 48 h. At 98 h increased expression of TGF-β, accompanied by loss of E-cadherin and acquisition of α-smooth muscle actin, was observed. The silence of ACC2 in HK-2 cells led to restored cell morphology with less lipid deposition and less malonyl-CoA content, which resulted from faster β-oxidation rate [33]. In another study, Xin et al. showed that palmitate-induced autophagy mediated by ACC2 in HK-2 cells and that palmitate-induced autophagy could be reduced ACC2 suppression [34]. In a mouse model with knock-in Serine to Alanine mutation of the phosphorylation sites of ACC, significant lipid accumulation and fibrosis were observed in tubular cells as compared to wild-type mice [35]. However, it was unclear if the KO of the enzyme would mitigate renal cell damage and fibrosis, nor is it clear how glycemic alteration would contribute to DNL. ACC is a a key regulatory enzyme in DNL, and its activity is under the hormonal influence of insulin, glucagon, and epinephrine. Specifically, insulin stimulates dephosphorylation and activation of ACC [36]. It is postulated that in insulin resistance such as type 2 diabetes, excess insulin may in part be responsible for enhanced ACC activity [12]. However, our study clearly shows that hyperglycemia can upregulate DNL in renal cell lines. Upregulation of DNL leading to abundance of palmitate has a flurry of devastating effects on renal cell lines that include activation of AMP-activated protein kinase and mammalian target of rapamycin complex-1 signaling pathways, insulin resistance, enhanced mitochondrial superoxide generation and endoplasmic reticulum stress, podocyte actin cytoskeleton impairment, promoted autophagy, apoptosis and eventually cell death [22,23,24,25,26,27,28,29], and hence our findings of decreased expression of markers of fibrosis and apoptosis (*Fas*) with DNL downregulation aliens logically with this pathophysiology. In our study downregulation of DNL by KO of either *Acaca* or *Acacb* genes in podocytes and tubular cells suggest that both isoforms are expressed in these cell lines and that they likely carry some degrees of overlapping function in DNL despite the specificity of ACC2 for carnitine palmitolytransferase-1 inhibition as a mitochondrial-membrane bound enzyme. 

*Srebp-1c* is a transcriptional factor that activates gene encoding enzymes required for fatty acid synthesis in the liver [37,38]. Kim et al. showed that double knocking out of ACC1/2 in the liver increased the expression of *Screpb-1c* and led to significant activation of *Gpat1*, the first committed step in triglyceride synthesis in the liver [39]. Upregulation of *Srebp-1c* also increased expression of fatty acid synthesis gene encoding enzymes including *Acly, Fas, Elovl6, Scd1, Pltp*, and *Pnpla3*; and led to significantly higher circulating triglycerides. This finding is consistent with the hypertriglyceridemic effect of ACC blockade reported in human clinical studies [40,41,42,43] which may be explained by activating effect *Srebp-1c* on *Gpat1* and increased incorporation of circulating fatty acids into VLDL triglycerides [39]. In our study, knocking out ACC encoding genes or pharmacological ACC blockade did not have any impact on the expression of *Srebp-1c* or *Gpat1* in renal cell lines and in fact led to lower expression of lipid synthesis markers *Scd1, Elovl6*, and *Acly*. These findings suggest that, unlike hepatocytes, tubular cells and podocytes are not equipped with triglyceride synthesis apparatus, consistent with a prior report that suggested renal accumulation of triglycerides is due to systemic level of fatty acids [44]. The clinical implication is that interventions specifically targeting renal ACC blockade might not be complicated by systemic hypertriglyceridemia. 

Our findings highlight important pathophysiological processes in podocytes and tubular epithelial cell lines. It reveals that hyperglycemic stress independently promotes upregulation of DNL which can have devastating consequences in tubular cells and podocytes which are not specialized to be the primary site for DNL. Common wisdom indicates that optimal control of glycemic stress should be the logical approach to achieving optimal DNL regulation in the kidney. To that end, insulin therapy is the core therapeutic approach to achieve this goal in type 1 diabetes and is widely used in type 2 diabetes as well. However, earlier studies have shown that despite beneficiary glycemic lowering effects, insulin also promotes activation of ACC via its dephosphorylation site [36]. Therefore, while insulin therapy continues to remain the core of the therapeutic approach in diabetes, ACC dysregulation due to glycemic stress or unwanted side effects of insulin abundance should be resolved for optimal outcomes. In this study, we showed that ACC inhibition effectively halted the upregulation of DNL and that the intervention was coupled with decreased makers of fibrosis and programmed cell death. These findings provide the scientific basis for testing their beneficial effects in further and future in vivo and clinical studies without which its reno-protective clinical potentials remain untested. 

This study also has limitations. This study is exclusively in vitro and in cultured immortal podocytes and in proximal tubular epithelial cells and hence cannot easily be translated into clinics. This provides proof of concept and the basis for further and future research which will require assessing the effect of DNL downregulation on the preservation of renal function and ultra-structures in vivo and eventually renal outcomes in clinical settings. In KO cells there is potential for residual ACC action due to partial efficacy of the KO process or the presence of a small fraction of wild-type cells allowing the synthesis of palmitate over time (Figure 2G). In cultured cells, there is also a potential for cellular transformation such as alteration of tubular cells to fibroblasts. However, in our study, the immunohistochemistry staining confirmed the cell markers specific to podocytes and tubular cells. Furthermore, the relatively short duration of observation up to 72 h makes the potential for cellular transformation quite low. Metabolic alterations in cell lines and specifically upon cellular transformation are another concern. In this study, we have utilized various cell lines that not only included immortalized cells but also primary tubular epithelial cells which are metabolically identical to their counterparts in vivo. Despite that, we observed highly reproducible and identical findings independent of the cell types, a convergence in findings which can be viewed as overall validity of the results. 

## 4. Materials and Methods

### 4.1. Isolation and Culture of Primary Tubular Epithelial Cells

Mouse primary tubular epithelial cells were isolated and cultured using the previously published protocol [45] with modification for mice. Briefly, mice (C57BL/6) were sacrificed and underwent cardiac perfusion with PBS. Kidneys were removed and injected with 0.5 mL of Krebs buffer, collagenase type II (1.0 mg/mL), 5 mM glycine, 5 mM glucose, 4.5 mM lactic acid, and heptanoic acid (2 mM). Cortex was separated, minced, and digested in the above Krebs buffer supplemented with 5 mM Mannitol and 2.5 mg/mL fatty acid-free BSA. The digestion is gentle shaking in 2 mL buffer, gassed with 95% O_2_/5% CO_2_, at 37 °C, for 10–15 min. The reaction was stopped by adding ice-cold Krebs buffer (2 × original volume) in a 15 mL conical tube and allowing clumps to settle for 1 min. The supernatant containing tubular cells was transferred to new 15 mL conical tubes and centrifuged at 30× *g* for 3 min. The pellet was suspended in 5 mL of BSA (2.5 mg/mL in cold Krebs buffer) and centrifuged again at 30× *g* for 3 min. The pellet was resuspended in 4.5 mL of 41% Percoll solution (41% *v*/*v* stock solution in Krebs buffer and 5 mg/mL BSA) and centrifuged at 37,000× *g* for 20 min at 4 °C. The tubule containing layer was transferred into new 15 mL conical tube and washed with wash buffer (by adding 5 mL of wash buffer containing 2 mg/mL gelatin in cold Krebs buffer and centrifugation at 30× *g* for 3 min) and plated in growth media (DMEM-F12 with 10% FBS, 1% penicillin/streptomycin, Insulin 5 ug/mL, transferrin 5 ug/mL, selenium 5 ng/mL, hydrocortisone 50 nM,) and an equal number of cells were plated.

### 4.2. Mouse Proximal Tubular Cells

Boston University Mouse Proximal Tubule (BUMPT) cells (BUMPT-Clone 306) were grown at 37 °C in DMEM F12 with 10% FBS as reported previously [46]. 

### 4.3. Mouse Podocytes

Mouse podocytes created by Dr. Mundel [47] were a gift from Drs. Bitzer and Inoki (Division of Nephrology, University of Michigan). They were grown in collagen-coated plates with RPMI media containing 10% Fetal Bovine Serum, 1% Insulin-Transferrin-Selenium (ITS), 1% penicillin-Streptomycin, and 100 U/mL Interferon Gamma. Cells were grown at 33 °C (permissive condition) and after reaching 60–70% confluency they were transferred to 37 °C (non-permissive condition) for differentiation in RPMI containing 10% FBS, 1% penicillin-Streptomycin, and 100 U/mL ITS without interferon gamma. 

### 4.4. Silencing Acaca and Acacb Genes with Different Methods

#### 4.4.1. CRISPR-Cas9 Mediated Knockout of Acaca and Acacb

Cells were transfected with *Acaca* and *Acacb* specific CRISPR-Cas9 plasmid using an electroporation technique following the manufacturer’s protocol and the corresponding RNA guide sequence (Supplementary Material Table S1). A double knockout of *Acaca* and *Acacb* together was also achieved by transfecting cells with both plasmids. The efficiency of knocking down was also verified with western blot analysis. The knockout cells along with controls were incubated in respective experimental media for different time points. 

#### 4.4.2. RNA Interference (Antisense LNA GapmeR) Mediated Acaca and Acacb Silencing

In Antisense LNA GapmeR experiments, overnight serum-starved cells were treated with specific GapmeR against either *Acaca* or *Acacb* (experimental group) or negative control GapmeR or with no GapmeR (control groups) in experimental media containing 25 mM glucose and 10 mM of ^13^C_2_-sodium acetate for different time periods. Fresh media containing 25 mM glucose and 10 mM labeled acetate was added to existing media every 24 h allowing GapmeR to present throughout the experimental time periods. The RNA guide sequence is shown in Table S1.

### 4.5. Relative Expression of mRNA Marker Genes by qPCR

Total RNA was isolated from the cells using RNeasy plus mini kts (Qiagen), according to the manufacturer’s protocol. RNA was reverse-transcribed with High-Capacity cDNA reverse transcription kit (Applied Biosystems, Waltham, MA, USA). Real-time qPCR was performed with qPCR primers (Table S1) using a Radiant SYBR green qPCR kit (Alkali Scientific Inc., Pompano Beach, FL, USA) in a Bio-Rad CFX Opus 384 system (Herculies, CA, USA). Relative mRNA abundance was normalized to the internal standard beta Actin with the ΔΔCT method.

### 4.6. Pharmacological Inhibition of ACC

We used firsocostat (ND 630) an ACC1 and ACC2 inhibitor. Experimental groups included overnight serum-starved cells that received fresh media containing 25 mM glucose and 10 mM of ^13^C_2_-sodium acetate and various doses of ACC inhibitor. Control cells received media with 25 mM glucose with 10 nM labeled acetate and no inhibitor. The cells were incubated for different time points with media replenishment every 24 h. 

### 4.7. Palmitate Labeling with Labeled Acetate

Cells were plated in 12-well plates containing 1 mL of specific growth media till reaching 70% confluency. The cells were serum starved overnight in respective serum-free media containing 5.5 mM glucose, 10 uM heptanoic acid (to mitigate the metabolic changes in the absence of serum), and 1% penicillin/streptomycin. After the incubation period, one set of cells (in triplicates) received fresh media containing 25 mM glucose and 10 mM of ^13^C_2_-sodium acetate, 1% penicillin/streptomycin, and 10 uM heptanoic acid and the other group received media with 5.5 mM glucose, 10 uM heptanoic acid, 19.5 mM Mannitol (to maintain identical osmolarity as in high glucose group) and 10 mM ^13^C_2_-sodium acetate (in addition to other growth components and antibiotics). Cells were incubated for different time points with media replenishment every 24 h. At the end of incubation, the media was removed, and the cells were washed with freshly prepared 150 mM ammonium acetate. The reaction was quenched by adding liquid nitrogen. Plates were then stored at −80 °C until further analysis.

### 4.8. Malonyl-CoA Assay

Cells for malonyl Co-A assay were prepared as mentioned above. After reaching 70% confluency, cells were serum starved overnight in low glucose (5.5 mM) media containing heptanoic acid. After incubation, media was changed to high glucose (25 mM) serum-free media with heptanoic acid, 10 mM of ^13^C_2_-sodium acetate, and incubated for different time periods with replenishment every 24 h. At the end of incubation, the reaction was stopped by removing the media and washed once with PBS. Cells were lysed in ice-cold PBS by a brief sonication followed by 2 freeze-thaw cycles. The lysate was centrifuged at 5000× *g* for 5 min at 4 °C. The supernatant was transferred to the fresh tube and protein concentration was measured. An equal amount of total protein was used for malonyl CO A assay using a mouse malonyl Co-A ELISA kit following the manufacturer’s protocol. Briefly, standards and samples were pipetted into the wells of a microplate pre-coated with antibodies specific for malonyl Co-A. After removing any unbound substances, a biotin-conjugated antibody specific for malonyl Co-A was added to the wells. After washing, avidin conjugated Horseradish Peroxidase (HRP) was added to the wells. Following a wash to remove any unbound avidin-enzyme reagent, a substrate solution was added to the wells, and color developed in proportion to the amount of malonyl Co-A bound in the initial step which was then measured. 

### 4.9. Immunofluorescence of Cell-Specific Markers

Immunofluorescence studies were done following standard established procedures. Briefly, cells were grown on sterilized, collagen-coated coverslips. Once they reached 60–70 confluency, cells were permeabilized using 0.1% Triton X 100 and fixed by incubating in CB buffer for 10 min at room temperature. Fixed cells on coverslips were washed with PBS and nonspecific sites were blocked using donkey serum and incubated with Alexa flour conjugated antibody against Neph 1 (for podocyte) and Megalin (for tubular epithelial cells) prepared in donkey serum overnight followed by washing with PBS. After washing coverslips were mounted onto microscope slides and left at 4 C in dark. Images were taken using a Leica microscope at different magnifications (Figure 1A,B).

### 4.10. Immunoprecipitation and Western Blot Analysis

Cell lysates were prepared using modified RIPA buffer containing protease inhibitors. The lysates were sonicated briefly followed by two freeze-thaw cycles centrifuged at 10,000× *g* for 10 min at 4 °C. Supernatant was collected and protein concentration was measured. Target proteins were immunoprecipitated and Western blot analyses were done using the manufacturer’s protocol. In brief, lysate with an equal amount of protein was precleared using control IgG for 30 min at 4 °C. The supernatant was incubated with agarose conjugated antibodies against ACC1, ACC2, and Fibronectin, with end over end rotation overnight at 4 °C. The pellet was collected by centrifugation and washed with PBS and resuspended in electrophoresis sample buffer along with sample reducing buffer and boiled for 5 min. The samples were separated on a gradient gel (4–12%) gel along with IgG and bead controls and transblotted to nitrocellulose membrane overnight at 30 V. The blots were blocked using 5% BSA in TBS and then incubated with target antibodies overnight followed by incubation with HRP conjugated secondary antibodies. Protein expression was visualized using ECL. The bands were quantified using the ImajeJ (NIH) program. 

### 4.11. Lipid Extraction, Liquid Chromatography, and Mass Spectrometry

We applied a modification of the Bligh–Dyer method [48] for lipid extraction. In brief, after bringing the quenched podocytes from −80 °C to room temperature, methanol/water/dichloromethane was added with a 2:2:2 ratio, followed by a collection of the organic layer and drying under nitrogen. Next, we reconstituted the samples by adding 100 μL of buffer B composed of acetonitrile/water/isopropyl alcohol (10:5:85 *v*/*v*/*v*) and 10 mM ammonium acetate. We further diluted the samples using Buffer B by 98% (sample:Buffer B, 2:98), and performed liquid chromatography (LC) separation on a Nexera X2 system (Shimadzu, Tokyo, Japan), with the autosampler temperature set to 10 °C. Then 5 μL of the sample was injected onto a reverse phase Acquity HSS T3 C_18_ column (50 mm × 2.1 mm, 1.8 μm, Waters, Milford, MA, USA) with the corresponding guard column (5 mm × 2.1 mm, 1.8 μm). Buffer A for liquid chromatography was prepared as acetonitrile:water (40:60 *v*/*v*). For separation, the flow rate was set to 0.4 mL/min, and the gradient was ramped linearly from 40% B to 98% B within 10 min, held at 98% B for 2 min, then ramped back to 40% B, and held at 40% B for 3 min. The eluents from LC were introduced into an AB Sciex Triple Quadrupole (/QTRAP 6500+) mass spectrometer (QTRAP 6500+ system, AB Sciex, Concord, Toronto, ON, Canada), and ions were via IonDrive Turbo V Ion Source (electrospray-ionization source) then measured in the negative ion mode through selected-ion-monitoring (SIM) with 100 ms dwell time for each ion monitored. The source voltage was set to 4500 V, the declustering potential was at −60 V, the entrance potential was at 10 V, and the source temperature was set to 550 °C. The curtain gas, nebulizer gas (gas 1), and turbo-gas (gas 2) were set as 35, 60, and 60 psi, respectively. We quantified the ratio of labeled palmitate (C16+2, m = 258.4 g/mol) over palmitate (C16, m = 256.4 g/mol) for downstream analysis. 

### 4.12. Statistical Analysis

To compare the mean values of the two study groups we used a t-test for independent samples. To compare the mean values of more than two study groups we used analysis of variance with Dunnett’s post hoc analysis to adjust for multiple comparisons and identify groups with significant differences compared with the reference group. The mass spectrometry experiments were repeated 3 times each time in triplicates. The immunoprecipitation assays were performed twice per condition, Malonyl CoA experiments three times, and qPCR experiments were also performed three times. All analyses were performed using IBM SPSS Statistics version 27 (Chicago, IL, USA). 

## 5. Conclusions

We conclude that glycemic stress can upregulate DNL in podocytes and tubular epithelial cells in vitro, and that pharmacological downregulation of ACC encoding genes or ACC inhibition can halt or mitigate the hyperglycemic induced upregulation of DNL in these cell lines a process that is coupled with decreased markers of fibrosis and programmed cell death. Further investigation is required to establish the protective effect of ACC inhibition in vivo and in clinical settings on renal outcomes.

## Data Availability

All the data are available at the possession of corresponding author and can be provided upon request. The data are not publicly available due to small sample size allowing visual track of the data points in manuscript’s graphs.

## Supplementary Materials

The following supporting information can be downloaded at: https://www.mdpi.com/article/10.3390/metabo12100940/s1, Table S1: The list of guide sequence RNA used in the manuscript; Materials S1.
