# Supplementary material for: Acetyl Co-A Carboxylase Inhibition Halts Hyperglycemia Induced Upregulation of De Novo Lipogenesis in Podocytes and Proximal Tubular Cells
Source: Metabolites. 2022 Oct 3;12(10):940. doi: 10.3390/metabo12100940 (PMC9610518; doi:10.3390/metabo12100940)
Supplement: Supplementary file 1 [file metabolites-12-00940-s001.zip › metabolites-1734781-supplementary.pdf]

Supplement:

Table S1: The list of guide sequence RNA used in the manuscript

Marker		Sequence
sc-430719: Accå CRISPR/Cas9 KO Plasmid (m) is a pool of 3 different gRNA plasmids:	sc-430719 A:	GCAGAATTTGTTACTCGTTT
	sc-430719 B:	AATGCATGCGATCTATCCGT
	sc-430719 C:	AAGTGTATCTGAGCTGACGG
sc-430335: Acc∫ CRISPR/Cas9 KO Plasmid (m) is a pool of 3 different gRNA plasmids:	sc-430335 A:	CGTGGTCATGGTGACGCCCG
	sc-430335 B:	GATCAGTACGTCCCTGTCCC
	sc-430335 C:	CGACGGTGAAATCTCTGTGC
GapmeRs	ACC negative control 5'-3'	AACACGTCTATACGC
	ACC alpha 5'-3'	TTTAGCGTTGGTGGTC
	ACC beta 5'-3'	CGAAAGAGCCGATTTG
Tgfb1	Forward, 5'-3'	TGATACGCCTGAGTGGCTGTCT
	Reverse, 3'-5'	CACAAGAGCAGTGAGCGCTGAA
Col1a1	Forward, 5'-3'	CCTCAGGGTATTGCTGGACAAC
	Reverse, 3'-5'	CAGAAGGACCTTGTTTGCCAGG
Col3a1	Forward, 5'-3'	GACCAAAAGGTGATGCTGGACAG
	Reverse, 3'-5'	CAAGACCTCGTGCTCCAGTTAG
col6A1	Forward, 5'-3'	TGCCCTGTGGATCTATTCTTCG
	Reverse, 3'-5'	CTGTCTCTCAGGTTGTCAATG
Srebp1c	Forward, 5'-3'	ATCGGCGCGGAAGCTGTCGGGGTAGCGTC
	Reverse, 3'-5'	ACTGTCTTGGTTGTTGATGAGCTGGAGCAT
Acly	Forward, 5'-3'	TTCGTCAAACAGCACTTCC
	Reverse, 3'-5'	ATTTGGCTTCTTGGAGGTG
Fas	Forward, 5'-3'	ATGCACACTCTGCGATGAAG
	Reverse, 3'-5'	CAGTGTTCACAGCCAGGAGA
Elovl6	Forward, 5'-3'	CGGCATCTGATGAACAAGCGAG
	Reverse, 3'-5'	GTACAGCATGTAAGCACCAGTTC
Scd1	Forward, 5'-3'	GCAAGCTCTACACCTGCCTCTT
	Reverse, 3'-5'	CGTGCCTTGTAAGTTCTGTGGC
Gpat1	Forward, 5'-3'	CAACACCATCCCCGACATC
	Reverse, 3'-5'	GTGACCTTCGATTATGCGATCA
G6pd	Forward, 5'-3'	CATCACCCTGGTACAACTCTT
	Reverse, 3'-5'	CGGCAACTAAACTCAGAAAAC
Pltp	Forward, 5'-3'	CCTAGCAAGACCTCTGCCAGTA
	Reverse, 3'-5'	GGACAGAAGGTTGGAGCCACAA
Pnpla3	Forward, 5'-3'	ACGTGCTGGTGTCTGAGTTCC
	Reverse, 3'-5'	AGGGACGTTGTCGCTCACTC
Dgpt1	Forward, 5'-3'	ACCGCGAGTTCTACAGAGATTGGT
	Reverse, 3'-5'	ACAGCTGCATTGCCATAGTTCCCT
bactin	Forward, 5'-3'	CCAGCCTTCCTTCTTGGGTAT
	Reverse, 3'-5'	TGCTGGAAGGTGGACAGTGAG

Materials S1

General chemicals, reagents and other materials for cell culture were purchased from Fisher Scientific, USA. $^{13}C_2$-sodium acetate was from Sigma-Aldrich, St. Louis MO. Pre-coated cell culture plates for podocytes culture (BioCoat) were from Corning, Kennebunk ME. RIPA buffer (89901) was from thermo scientific, Rockford, IL complete protease inhibitor cocktail was from Sigma (R0278). Complete protease inhibitor cocktail was from Roche Diagnostic, Mannheim Germany (11836170001). Agarose conjugated antibodies against ACC1 (sc-137104), ACC2 (SC-390344 AC), Fibronectin (SC 59826 AC) for immunoprecipitation and Alexa flour 546 conjugated antibodies against Neph1 (sc-373787 AF 546) and Megalin (sc-515772 AF 546), fibronectin (sc-59826) as well as CRISPR/cas 9 plasmids for ACC1 (sc-430719), and ACC2 (sc-430335) were from Santa Cruz biotechnology, Dallas TX. Enhanced chemiluminecent substrate for HRP (ECL) was from Thermoscientific, Waltham, MA (32106). Antibody for ACC1 protein detection was from thermofisher (21923-AP); ACC 2 was from LS Bio, Seattle WA (LSC806941) and fibronectin from abcam, Waltham MA (ab2413). Gels and reagents for Western blotting was from Invitrogen (Sample buffer NP0007, reducing buffer NP0009, 4-12% gel NP0321). Malonyl Co-A ELISA kit (MBS705127) was from MYBioSource, San Diego, CA. AMAXA cell line nucleofector kit (VCA-1003) for electroporation was from Lonza, Cologne AG, Germany. Antisense LNA (GapmeR) and RNeasy plus RNA extraction kit (cat #74134) was purchased from Qiagen, Germantown, MD. cDNA reverse transcription kit (cat # 4374966) was from applied biosystem Grand Island NY and SYBR green qPCR kit (Qs 1005) was from Alkali Scientific Inc. Fort Lauderdale FL.
